# Association of multiple symptoms with sleep quality and duration: a cross-sectional population-based study of older men in Sweden

**DOI:** 10.1136/bmjopen-2024-094962

**Published:** 2025-07-01

**Authors:** Maria Zeaiter, Max Olsson, Slavica Kochovska, David C Currow, Magnus Ekström

**Affiliations:** 1 Lund University, Faculty of Medicine, Department of Clinical Sciences Lund, Respiratory Medicine, Allergology and Palliative Medicine, Lund, Sweden; 2 Faculty of Science, Medicine and Health, University of Wollongong, Wollongong, New South Wales, Australia; 3 Faculty of Health, University of Technology Sydney, IMPACCT, Ultimo, New South Wales, Australia

**Keywords:** Sleeping problems, dyspnea, breathlessness, pain, symptoms, symptom prevalence, population study

## Abstract

**Abstract:**

**Objective:**

To evaluate any association of the presence and severity of nine major symptoms (pain, tiredness, drowsiness, nausea, appetite, breathlessness, depression, anxiety, and perceived well-being) with sleep quality and duration in elderly men.

**Design and setting:**

Cross-sectional analysis within the population-based VAScular and Chronic Obstructive Lung disease study (VASCOL) conducted in southern Sweden in 2019.

**Participants:**

A total of 838 older men aged 73 years.

**Measures:**

Severity of the symptoms was self-reported between 0 and 10 on a numerical rating scale. Breathlessness was also assessed using the Multidimensional Dyspnoea Profile and Dyspnoea-12. Sleep quality was reported on a 5-point Likert scale from ‘very well’ to ‘very bad’and sleep duration on a 7-point scale from ‘less than 4 hours’ to ‘more than 10 hours’. Associations of each symptom score with having worse sleep quality (‘bad’ or ‘very bad’) and/or shorter sleep duration (<6 hour/night) were analysed using logistic regression, adjusted for common confounders.

**Results:**

Of 838 men analysed, 11% had worse sleep quality, 8% had shorter sleep duration and 5% had both. Higher severity of each symptom was associated with worse sleep quality (adjusted odds ratios (aOR) ranging 1.12–1.61) and shorter sleep duration (aORs ranging 1.10–1.49).

**Conclusions:**

A wide range of symptoms is associated with worse sleep quality and shorter sleep duration in elderly men, suggesting that clinicians should assess sleep when these symptoms are present and *vice versa*.

**STRENGTHS AND LIMITATIONS OF THIS STUDY**

- A range of clinically relevant symptoms were analysed and adjusted for potential confounders.
- Symptom severities were measured using validated instruments.
- The inclusion of only 73 year-old men effectively precludes age- or sex-related bias but decreases the generalisability of the findings.
- The statistical relationships between exposures and outcomes do not infer causality.

## Introduction

 Sleeping problems involve a disruption in sleep duration and/or quality^1^ and are highly prevalent,^2^ affecting up to 30% of the general population.^3^ Insufficient sleep quality and duration are associated with worse physical and mental states,^2^ lower work productivity^4^ and increased morbidity and mortality.^5 6^

Sleeping problems are caused by multiple factors including primary sleep disorders,^7^ behavioural and environmental causes,^7^ as well as underlying illnesses.^8 9^ Compared with younger people, older people have an increased prevalence of fragmented sleep with earlier awakening.^7^ Age-related changes in sleep have been associated with medical conditions such as cerebrovascular disease, cardiovascular disease, Alzheimer’s disease, chronic obstructive pulmonary disease and depression.^7^

Symptoms, such as breathlessness, fatigue, pain, anxiety and depression, are common among the elderly,^2 10 11^ are main reasons for seeking health care^12^ and are strongly related to worse health-related quality of life.^11 13 14^

Knowledge is incomplete as to whether, and to what degree, the presence and severity of symptoms are associated with sleeping problems. Depression^15^ and anxiety^16^ have been associated with sleeping problems, but without considering the severity of the symptoms. The association between the severity of breathlessness,^2^ fatigue^17^ and pain^18^ and sleeping problems has been suggested, but associations with sleep across a broader range of symptoms have not been thoroughly studied. Knowledge is also limited on any association of various symptoms on sleep quality and duration, independently of underlying comorbidities. Understanding this relationship is important due to the high prevalence and burden of these conditions in older people and the complexity of sleeping problems. Hypothetically, symptoms such as pain or breathlessness could contribute to worse sleep quality and shorter sleep duration, while sleeping problems could amplify any symptom. Studying the symptoms associated with sleeping problems independently of the underlying condition could lead to a deeper understanding of sleeping problems which, in turn, could contribute to better assessment and management of factors such as symptoms for improved sleep.

The primary aim of this study was to evaluate any associations between the severity of nine symptoms (pain, tiredness, drowsiness, nausea, appetite, breathlessness, depression, anxiety and overall well-being) with sleep quality and duration among older men in the community. Our secondary aim was to evaluate which symptom(s) had the strongest independent relation to sleep outcomes where all symptoms were analysed in the same model simultaneously, adjusted for confounders.

## Methods

### Design and population

This was a cross-sectional analysis of the VAScular and Chronic Obstructive Lung disease (VASCOL) study and a longitudinal study of morbidity, symptoms and quality of life among elderly men.^19^ The VASCOL database has been detailed elsewhere and used in previous analyses,^19^,^21^ but the present analyses are novel and have not been presented before.

All the methods in this study followed the Strengthening the Reporting of Observational studies in Epidemiology (STROBE) guidelines.^22^

Between 2010 and 2011, a random sample of 1900 out of all 2300 men aged 65 years in the county of Blekinge, Sweden, was invited to ultrasound screening for abdominal aortic aneurysms and asked to participate in the VASCOL study.^19^ A total of 68.5% (1,302/1,900) participated in the first assessment in 2010–2011, which included baseline characteristics but no patient-reported outcomes. Participants in the VASCOL baseline assessment had similar characteristics compared with age-matched men in the Swedish general population regarding everyday smoking, spirometry values, height and civil status, but had a higher proportion of former smokers, BMI and educational level.^19^

Between 1 March 2019 and 28 September 2019, 1193 (91.6%) participants (now aged about 73 years) who were still alive and had a known postal address were asked to complete a postal questionnaire on self-reported lifestyle factors, medical conditions, medications, sleep quality, sleep duration, and presence and severity of nine symptoms (pain, tiredness, drowsiness, nausea, appetite, breathlessness, depression, anxiety and overall well-being) during the participants' previous 2 weeks. The variables and assessments are detailed below.

Exclusion criteria for this analysis were missing data on self-reported sleep quality, sleep duration and any of the nine symptoms. No data were imputed.

### Assessments

Sleep quality was assessed by asking participants to rate how well they had slept in the last 2 weeks on a 5-point Likert scale (1=very well; 2=well; 3=quite well; 4=bad ; 5=very bad). Sleep duration was assessed by asking how many hours they usually slept per day during the last 2 weeks on a 7-point scale (1 = ≤4 hours; 2= 5 hours; 3= 6 hours; 4= 7 hours; 5= 8 hours; 6= 9 hours; 7 = ≥10 hours).

Symptom severities were self-reported using the revised Edmonton Symptom Assessment System (ESAS-r) for the nine symptoms: pain, tiredness, drowsiness, nausea, appetite, breathlessness, depression, anxiety and overall well-being.^23^ Each symptom severity was rated on a numerical rating scale (NRS) between 0 (none) and 10 (worst possible).^23^

Breathlessness was also assessed using the Multidimensional Dyspnoea Profile (MDP)^24 25^ overall unpleasantness (A1) score and the Dyspnoea-12 (D-12) total score.^26 27^ The MDP-A1 score is a 10-point rating scale measuring breathlessness unpleasantness between 0 (neutral) and 10 (excruciating).^24 25^ The D-12 total score is a sum of a 12-item questionnaire consisting of seven physical and five affective items of breathlessness, each scored between 0 (none) and 3 (severe).^26^ D-12 total ranges from 0 to 36, with higher scores indicating more severe breathlessness.^26^

### Statistical analyses

Patient characteristics were described. Association of sleep quality and duration, respectively, with each of the nine symptoms was assessed using box plots. Based on the distribution of the scores, sleep quality was categorised as better (very well; well; or quite well) or worse (bad; very bad). Sleep duration was categorised as shorter (<6 hours/night) or longer.

Associations were analysed using logistic regression models. A primary analysis was conducted between each of the nine symptoms of ESAS-r with sleep quality and duration, respectively. Estimates were analysed crude and adjusted for potential confounders and were expressed as ORs with 95% CIs. Confounders were selected based on subject matter knowledge and previous research: smoking, alcohol, cortisone tablets, ischaemic heart disease, atrial fibrillation, heart failure, valvular heart disease, bypass operation, stroke, chronic obstructive pulmonary disease, asthma, sleep apnoea, rheumatism, cancer, exertion, bronchial dilators and cortisone inhalers.^7^,^9^

All nine symptoms were then analysed in the same model simultaneously in relation to the outcomes, adjusted for potential confounders. For breathlessness, analyses were also performed using the MDP-A1 and D-12 total score.

Among the potential confounders, use of opioids and sedatives could not be included in the analysis due to a low prevalence (opioids, n=5; sedatives, n=21).

Associations were expressed as ORs with 95% CIs. As boxplots of sleep duration by symptom severity scores suggested U-shaped associations, we also conducted a linear regression model of the symptom severity as the dependent variable and sleep duration (independent variable) categorised into three categories of longer sleep duration: 1 (≤5 hours/night); 2 (6–8 hours/night) and 3 (≥9 hours/night). A two-sided p-value below 0.05 was considered as statistically significant. No data were imputed. All analyses were conducted using Stata, version 17.0 (StataCorp LP; College Station, TX; USA).

### Patient and Public Involvement

The survey was piloted on 10 people of similar age to the VASCOL study, which gave feedback on the survey. Minor layout changes were done to the survey questions to fit the specific study participants.^19^

### Ethical considerations

This study was approved by the Regional Ethical Review Board at Lund University (reference number: 2008/676) and the National Ethical Review Board (reference number: 2019–00134).^19^ All participants provided written informed consent.

## Results

### Participants’ characteristics

**Figure 1:**
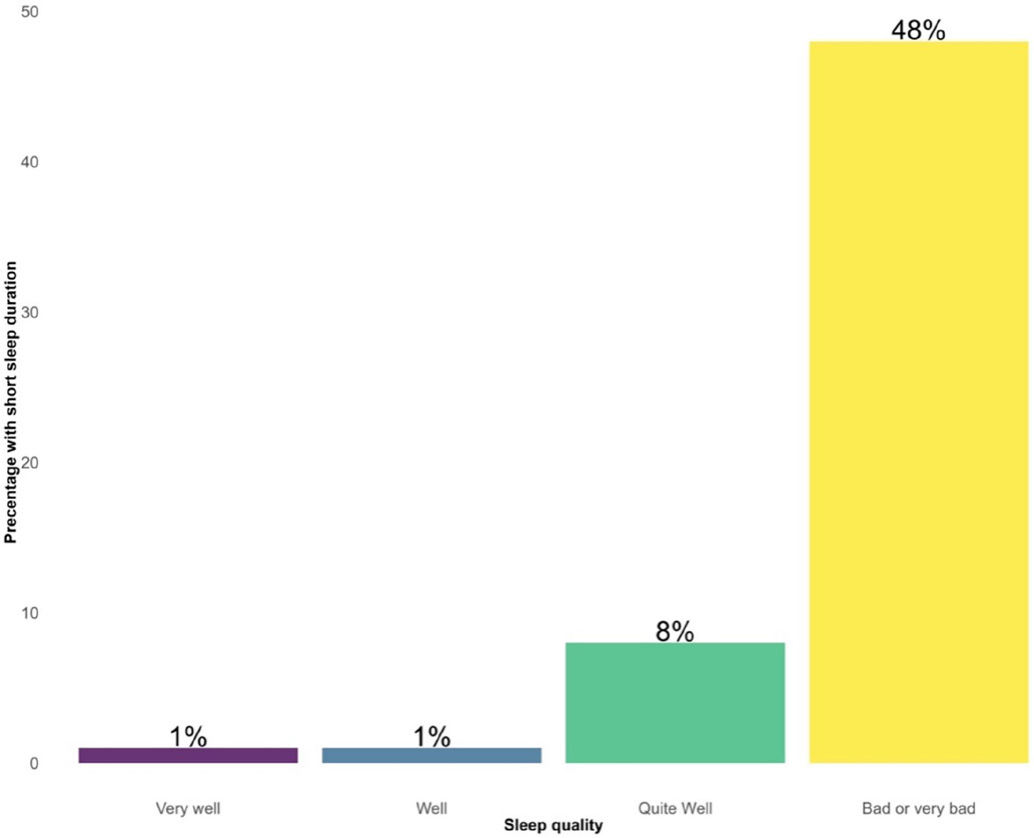
Association between sleep quality and short sleep duration in 838 men aged 73 years

Of the 907 men who participated in the survey in 2019, 69 were excluded because of missing data and the remaining 838 (92%) were included in the analysis. Among the included participants, 11% had worse sleep quality (n=88; table 1), 8% had shorter sleep duration (n=71; online supplemental table S1), while 5% (n=42) had both worse sleep quality and shorter sleep duration. Worse sleep quality was associated with a higher percentage of short sleep duration (figure 1).

**Table 1:** Participant characteristics

	Better sleep quality	Worse sleep quality	P value
N	750 (89%)	88 (11%)	
Age, mean (SD)	73.19 (0.65)	73.29 (0.70)	0.188
Smoking status			0.044
Daily	36 (4.8%)	5 (5.7%)	
Sometimes	8 (1.1%)	1 (1.1%)	
Former smoker	438 (58.4%)	62 (70.5%)	
Never smoked	262 (34.9%)	18 (20.5%)	
Alcohol consumption, standard units per week, mean (SD)	6.83 (7.01)	6.82 (7.75)	0.983
Ischaemic heart disease (angina, heart attack)	96 (12.8%)	16 (18.2%)	0.160
Atrial fibrillation	108 (14.4%)	19 (21.6%)	0.075
Heart failure	27 (3.6%)	6 (6.8%)	0.142
Valvular heart disease	33 (4.4%)	5 (5.7%)	0.585
Bypass operation	69 (9.2%)	8 (9.1%)	0.973
Stroke	60 (8.0%)	4 (4.5%)	0.248
Chronic obstructive pulmonary disease	23 (3.1%)	5 (5.7%)	0.197
Asthma	38 (5.1%)	7 (8.0%)	0.256
Sleep apnoea	54 (7.2%)	18 (20.5%)	<0.001
Rheumatism	33 (4.4%)	5 (5.7%)	0.585
Cancer	121 (16.1%)	13 (14.8%)	0.742
Exertion level			0.028
Sedentary	51 (6.8%)	10 (11.4%)	
Moderate	442 (58.9%)	57 (64.8%)	
Moderate but regular	199 (26.5%)	13 (14.8%)	
Frequent	44 (5.9%)	2 (2.3%)	
Inhaled bronchodilator(s)	58 (8.6%)	12 (15.2%)	0.055
Inhaled corticosteroids	42 (6.2%)	5 (6.6%)	0.896
Oral corticosteroids	19 (2.8%)	2 (2.7%)	0.946

Data are presented as frequency (percentage) or mean (standard deviation; SD). *Sleep quality was categorised as better (very well; well; or quite well) or worse (bad; very bad).*

Characteristics of the included participants are shown in table 1. Compared with participants with better sleep quality and longer sleep duration, participants in each category of worse sleep quality and shorter sleep duration were more likely to have cardiovascular and pulmonary diseases, smoke, exercise less and drink more alcohol (table 1; online supplemental table S1). The prevalence of stroke and cancer was higher in the groups with better sleep quality (8% vs 4.5% for stroke; 16.1% vs 14.8% for cancer; table 1) and longer sleep duration (7.7% vs 7% for stroke; 16.2% vs 14.1% for cancer) (online supplemental table S1).

### Sleep Quality

**Figure 2:**
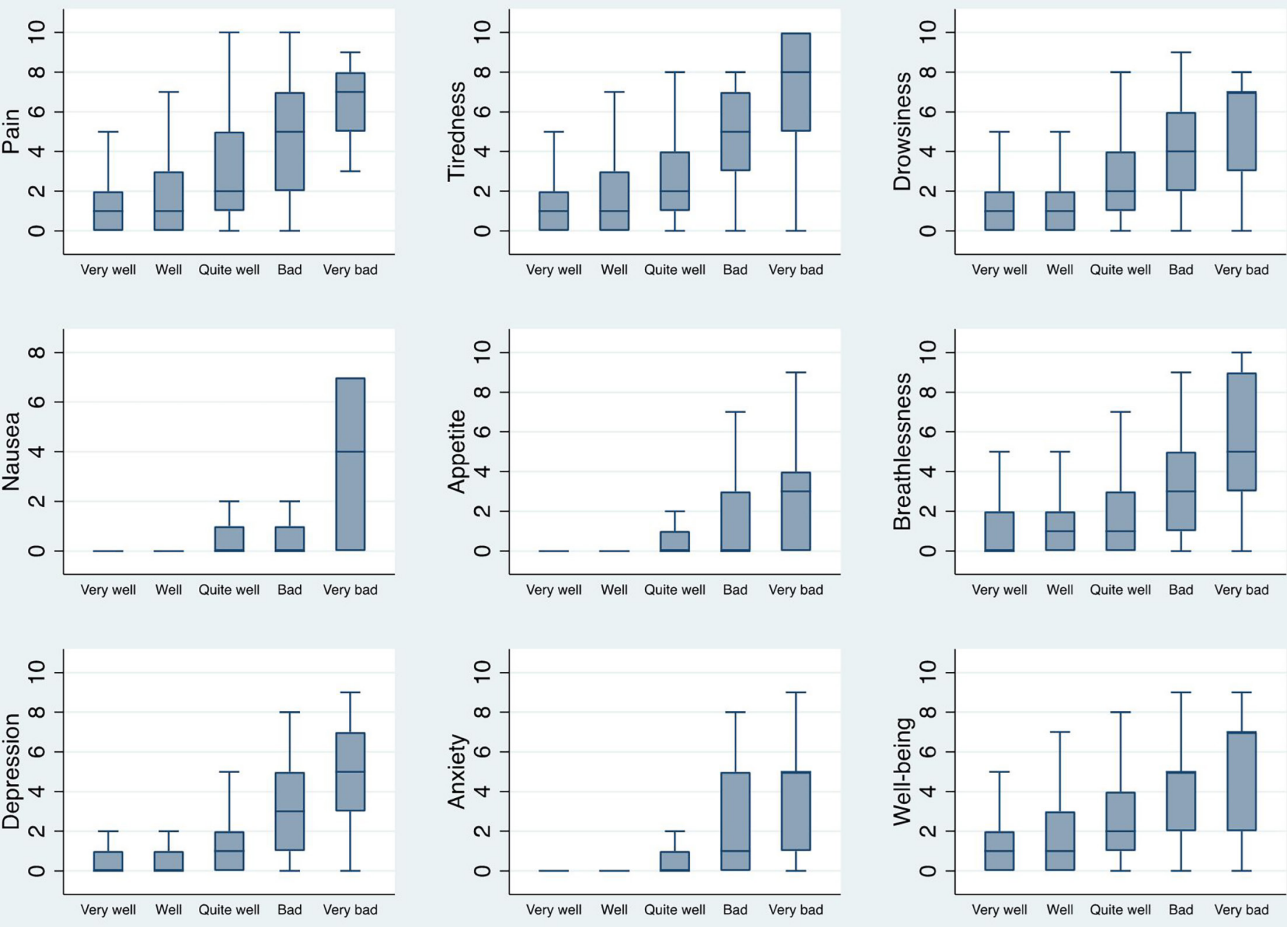
Symptom severities associated with sleep quality. Sleep quality was assessed by asking participants to rate how well they had slept in the last two weeks on a 5-point Likert scale as presented in the figure

Sleep quality worsened with greater symptom severity (figure 2, online supplemental figure S1). In the regression models, higher severity of each symptom was associated with worse sleep quality and the associations remained after adjusting for potential confounders (table 2).

**Table 2:** OR of worse sleep quality and shorter sleep duration by each symptom separately, adjusted for confounders.

Symptom	Worse sleep quality				Shorter sleep duration			
	Crude OR (95% CI)	Crude P value	Adjusted OR (95% CI)	Adjusted P value	Crude OR (95% CI)	Crude P value	Adjusted OR (95% CI)	Adjusted P value
Pain	1.43 (1.31 to 1.56)	<0.001	1.44 (1.27 to 1.63)	<0.001	1.33 (1.21 to 1.46)	<0.001	1.38 (1.21 to 1.58)	<0.001
Tiredness	1.50 (1.37 to 1.65)	<0.001	1.58 (1.38 to 1.82)	<0.001	1.42 (1.29 to 1.56)	<0.001	1.49 (1.29 to 1.72)	<0.001
Drowsiness	1.54 (1.40 to 1.69)	<0.001	1.61 (1.39 to 1.86)	<0.001	1.41 (1.28 to 1.55)	<0.001	1.43 (1.23 to 1.65)	<0.001
Nausea	1.51 (1.34 to 1.70)	<0.001	1.55 (1.29 to 1.86)	<0.001	1.39 (1.23 to 1.58)	<0.001	1.42 (1.18 to 1.70)	<0.001
Appetite	1.47 (1.32 to 1.64)	<0.001	1.36 (1.16 to 1.60)	<0.001	1.34 (1.20 to 1.50)	<0.001	1.29 (1.08 to 1.53)	0.016
Breathlessness	1.30 (1.20 to 1.41)	<0.001	1.24 (1.10 to 1.40)	<0.001	1.27 (1.16 to 1.38)	<0.001	1.29 (1.13 to 1.47)	<0.001
Depression	1.51 (1.37 to 1.65)	<0.001	1.44 (1.27 to 1.63)	<0.001	1.41 (1.29 to 1.56)	<0.001	1.40 (1.23 to 1.59)	<0.001
Anxiety	1.46 (1.33 to 1.61)	<0.001	1.43 (1.26 to 1.64)	<0.001	1.40 (1.27 to 1.54)	<0.001	1.38 (1.19 to 1.59)	<0.001
Well-being	1.37 (1.25 to 1.49)	<0.001	1.34 (1.20 to 1.50)	<0.001	1.24 (1.13 to 1.36)	<0.001	1.19 (1.05 to 1.35)	0.009
MDP-A1 score	1.45 (1.29 to 1.64)	<0.001	1.48 (1.21 to 1.82)	<0.001	1.38 (1.21 to 1.57)	<0.001	1.41 (1.14 to 1.75)	0.026
D-12 total score	1.13 (1.08 to 1.17)	<0.001	1.12 (1.05 to 1.20)	<0.001	1.10 (1.05 to 1.14)	<0.001	1.10 (1.02 to 1.18)	0.006

Adjusted OR for potential confounders (smoking, alcohol, cortisone tablets, ischaemic heart disease, atrial fibrillation, heart failure, valvular heart disease, bypass operation, stroke, chronic obstructive pulmonary disease, asthma, sleep apnoea, rheumatism, cancer, exertion, bronchia dilators and cortisone inhalators). Sleep quality was categorised as better (very well; well or quite well) or worse (bad or very bad). Sleep duration was categorised as shorter (< 6 hours/night) or longer.

D-12 total, Dyspnoea-12 total score; MDP-A1, Multidimensional Dyspnoea profile overall unpleasantness (A1) score; OR, odds ratio.

When analysing all the symptoms in the same model concurrently having adjusted for confounders, no symptom was clearly more associated than another, as indicated by the overlapping CIs between the symptoms (online supplemental table S2).

### Sleep duration

**Figure 3:**
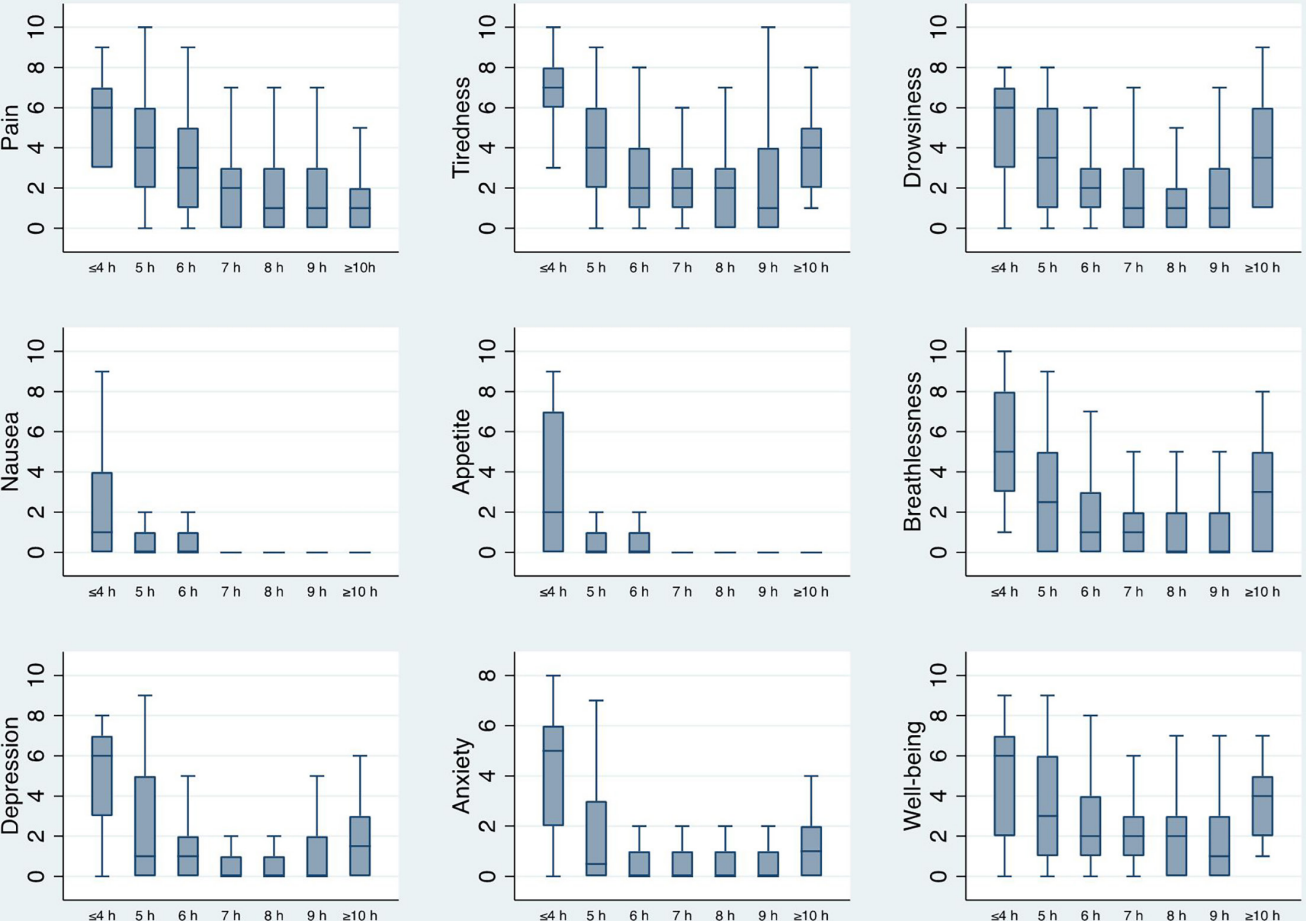
Symptom severities associations to sleep duration. Sleep duration was assessed by asking how many hours they usually slept per day during the last 2 weeks on a 7-point scale as presented in the figure

Sleep duration tended to associate with symptom severities in a U-shaped manner, that is, higher symptom scores were associated with people who had the shortest and, separately, the longest sleep durations (figure 3, online supplemental figure S1). However, the trend for U-shaped associations was not significant when analysed using three categories of sleep duration (low, middle, high) in linear regression (data not shown), with the 95% CIs for the estimates crossing zero and all p-values being >0.166 (comparing to the middle category). The associations between symptom severities and sleep duration were therefore analysed using the two previously selected categories and multiple logistic regression. Shorter sleep duration was associated with higher severity of each symptom, both in the unadjusted and adjusted models (table 2).

When analysing all symptoms concurrently, the association of symptom severity with shorter sleep duration was also overlapping in both the adjusted and unadjusted models (online supplemental table S2).

## Discussion

In this population-based study of 73-year-old men, the main finding is that having more severe symptoms was associated with having worse sleep quality and shorter sleep duration. This study supports previous observations that sleep problems may be associated with the severity of breathlessness,^28^ pain^29^and anxiety.^16^ The study also reports an association between sleep problems and six other symptoms, including tiredness, drowsiness, nausea, appetite, depression and well-being that have not been studied before. To our knowledge, this is the first population-based study to analyse the association between the severity of a range of major symptoms concurrently with sleep problems. We did not find associations between symptoms and sleep to be related to the set of evaluated potential confounding factors. This supports the importance of recognising symptoms as essential elements in sleep disturbances, independent of other factors such as underlying comorbidities.

Although this cross-sectional analysis cannot infer causality, it is likely that pain, nausea, worse appetite, breathlessness, anxiety and worse well-being probably contribute to worse quality and shorter duration of sleep, given our current understanding of the impact of symptoms on well-being and sleep. Lack of quality sleep can also worsen each of these symptoms. At the other end, although sleep and symptoms can worsen each other, we hypothesise that the main direction of influence is that sleeping problems contribute to drowsiness, tiredness and depression.

We also evaluated all symptoms concurrently in the same model (controlling each analysis for the severities of all other symptoms and factors in the analysis) in order to explore which symptom had the strongest association to worse sleep independent of the other (concurrent) symptom levels. This novel analysis could not identify any of the symptoms that were more strongly related to worse sleep outcomes, as the CIs were largely overlapping. We acknowledge that the manner in which the different symptoms interplay is complex^30^ and suggest that symptoms have similar and, perhaps to an extent, common pathways in the brain that are complicated and heterogenic and are yet to be studied and identified.

Our finding that symptom severity is associated with shorter sleep duration is contrary to previous studies indicating that sleeping for ‘too long’ is associated with severe symptoms, including pain,^31^ depression^9 32^ and tiredness.^33^ Some studies have also reported that sleeping too long is also associated with severe diseases such as hypertension,^34 35^ cardiovascular diseases,^35^ diabetes mellitus type 2^35^ and increased mortality.^36^ Hence, a study with a larger sample size may help to further elucidate any relationship.

One interesting finding is that participants who had stroke and cancer slept better and longer, although the differences were not significant. Some studies have shown a significant association between stroke and hypersomnia.^37 38^ However, several studies have shown a common prevalence of sleep problems including hypersomnia and insomnia in cancer patients,^39 40^ with contributing factors including cancer treatment and cancer-related psychological aspects.^39^

Strengths of this study include the analysis of a range of clinically relevant symptoms and potential confounders. Symptom severities were measured using validated instruments. Limitations include the retrospective, cross-sectional design. The statistical relationships between exposures and outcomes do not infer causality. The inclusion (in relation to an aortic aneurysm screening programme) of only 73-year-old men effectively precludes age- or sex-related bias, but may decrease the generalisability of the findings. Importantly, these findings should be validated in more diverse populations including women. The cohort was overall healthy with low prevalence of chronic diseases and is comparable with older males in Sweden,^19^ but the generalisability of our findings could be worse for populations with a higher prevalence of chronic diseases. People with chronic diseases are probably less likely to participate in studies, and there is therefore a risk that we underestimated the burden of worse sleep quality and short sleep duration in the older general population. Compared with previous studies of sleep quality among older people,^7 41^ our study had a low prevalence of participants with sleep problems that could not only be explained by our overall healthy cohort^19^ but also by different measurements and definitions of worse sleep quality. Although several evaluated confounding factors were adjusted in the models, residual confounding could still be present, such as genetics, intake of illegal drugs or bad sleep hygiene. Sleep was measured using self-reported assessments only, which could be affected by respondents’ feelings and thoughts prompting exaggerated response or potential embarrassment to reveal personal details. Another limitation might be missing data on sleep medications, which could contribute to underestimating the associations as some people with sleep problems may use medications to treat their sleep problems. However, it might not play a major role since we studied the associations between different symptoms measured in the same participants.

The current findings suggest that physical symptoms are associated with worse sleep. This is relevant for clinical practice, as is the suggestion that improved symptomatic treatment could be an effective way to improve sleep in older people.

The current findings and the temporal relationships between symptoms and sleeping problems should be evaluated using longitudinal data. Future controlled trials should compare the outcome of a control group to a group where anamnesis of different symptoms is penetrated and treated. Further insights on how different physiological pathways regarding symptoms interact in the brain would help establish a deeper understanding of treating sleeping problems.

### Conclusion

In conclusion, in people with a range of symptoms, clinicians should ask about quality and duration of sleep. In people with poor sleep quality or short duration, clinicians should inquire about long-term symptoms.

## Supplementary material

10.1136/bmjopen-2024-094962 online supplemental material 1

## Data Availability

Data are available upon reasonable request.
