# Supplementary material for: Association of multiple symptoms with sleep quality and duration: a cross-sectional population-based study of older men in Sweden
Source: BMJ Open. 2025 Jul 1;15(7):e094962. doi: 10.1136/bmjopen-2024-094962 (PMC12215098; doi:10.1136/bmjopen-2024-094962)

**SUPPLEMENTAL MATERIAL**

**Table S1:** Participant characteristics in the follow-up survey by shorter and longer sleep duration.

|  | **Longer sleep duration** | **Shorter sleep duration** | **p-value** |
| --- | --- | --- | --- |
| N | 767 (92%) | 71(8%) |  |
| Age | 73.20 (0.66) | 73.20 (0.69) | 0.96 |
| Smoking  Daily  Sometimes  Former smoker  Never smoked | 38 (5.0%)  8 (1.0%)  454 (59.2%)  261 (34.0%) | 3 (4.2%)  1 (1.4%)  46 (64.8%)  19 (26.8%) | 0.56 |
| Standard units alcohol | 6.84 (6.89) | 6.73 (9.12) | 0.91 |
| Ischemic heart disease (angina, heart attack | 101(13.2%) | 11(15.5%) | 0.58 |
| Atrial fibrillation | 113 (14.7%) | 14 (19.7%) | 0.26 |
| Heart failure | 30 (3.9%) | 3 (4.2%) | 0.90 |
| Valvular heart disease | 34 (4.4%) | 4 (5.6%) | 0.64 |
| Bypass operation | 74 (9.6%) | 3 (4.2%) | 0.13 |
| Stroke | 59 (7.7%) | 5 (7.0%) | 0.84 |
| Chronic Obstructive Pulmonary Disease | 25 (3.3%) | 3 (4.2%) | 0.66 |
| Asthma | 37 (4.8%) | 8 (11.3%) | 0.021 |
| Sleep Apnoea | 61 (8.0%) | 11 (15.5%) | 0.03 |
| Rheumatism | 34 (4.4%) | 4 (5.6%) | 0.64 |
| Cancer | 124 (16.2%) | 10 (14.1%) | 0.65 |
| Exertion  Sedentary  Moderate  Moderate but regular  Frequent | 51 (6.6%)  460 (60.0%)  199 (25.9%)  44 (5.7%) | 10 (14.1%)  39 (54.9%)  13 (18.3%)  2 (2.8%) | 0.079 |
| Bronchial dilators | 60 (8.7%) | 10 (15.4%) | 0.075 |
| Inhaled corticosteroids | 42 (6.1%) | 5 (7.9%) | 0.56 |
| Oral corticosteroid treatment | 20 (2.9%) | 1(1.6%) | 0.56 |

Data presented as n(%) or mean (standard deviation).

**Table S2:** Odds ratio of worse sleep quality and shorter sleep duration by all symptoms concurrently, adjusted for confounders.

|  | **Worse sleep quality** | **Shorter sleep duration** |
| --- | --- | --- |
| **ESAS-r** | **aOR*(95%CI)** | **aOR*(95%CI)** |
| Pain | 1.14(0.96-1.35) | 1.16(0.97-1.40) |
| Tiredness | 1.18(0.90-1.54) | 1.28(0.97-1.68) |
| Drowsiness | 1.26 (0.99-1.61) | 1.02(0.80-1.30) |
| Nausea | 1.24 (0.96-1.61) | 1.14(0.88-1.49) |
| Appetite | 0.88 (0.68-1.12) | 0.87(0.66-1.14) |
| Breathlessness | 0.91 (0.77-1.07) | 1.03(0.87-1.22) |
| Depression | 1.01 (0.77-1.32) | 1.21(0.91-1.61) |
| Anxiety | 1.09 (0.85-1.40) | 1.04(0.79-1.37) |
| Well-being | 1.12 (0.95-1.34) | 0.85(0.68-1.08) |
| **ESAS-r + MDP-A1** |  |  |
| Pain | 1.14(0.95-1.36) | 1.20(0.99-1.46) |
| Tiredness | 1.10(0.84-1.43) | 1.28(0.98-1.67) |
| Drowsiness | 1.24(0.97-1.59) | 1.00(0.79-1.29) |
| Nausea | 1.21(0.93-1.58) | 1.15(0.88-1.50) |
| Appetite | 0.87(0.68-1.12) | 0.84(0.64-1.12) |
| MDP-A1 | 1.06(0.84-1.34) | 1.10(0.85-1.42) |
| Depression | 1.01(0.78-1.32) | 1.22(0.91-1.63) |
| Anxiety | 1.11(0.86-1.42) | 1.04(0.79-1.38) |
| Well-being | 1.13(0.95-1.35) | 0.85(0.67-1.08) |
| **ESAS-r + D-12 total** |  |  |
| Pain | 1.16(0.97-1.39) | 1.17(0.97-1.40) |
| Tiredness | 1.11(0.85-1.45) | 1.27(0.98-1.66) |
| Drowsiness | 1.25(0.98-1.60) | 1.02(0.80-1.30) |
| Nausea | 1.25(0.96-1.63) | 1.13(0.86-1.48) |
| Appetite | 0.87(0.67-1.12) | 0.87(0.66-1.14) |
| D-12 total | 1.00((0.92-1.09) | 1.00(0.92-1.10) |
| Depression | 0.97(0.74-1.27) | 1.21(0.91-1.60) |
| Anxiety | 1.10(0.85-1.42) | 1.03(0.78-1.36) |
| Well-being | 1.16(0.96-1.39) | 0.87(0.68-1.10) |

aOR*: adjusted odds ratio for potential confounders (smoking, alcohol, cortisone tablets, ischemic heart disease, atrial fibrillation, heart failure, valvular heart disease, bypass operation, stroke, chronic obstructive pulmonary disease, asthma, sleep apnoea, rheumatism, cancer, exertion, bronchia dilators and cortisone inhalators). CI: confidence interval. ESAS-r: the revised Edmonton Symptom Assessment scale. D-12 total: Dyspnea-12 total score. MDP-A1: Multidimensional Dyspnea profile overall unpleasantness (A1) score.

**MM**

**Figure S1:** Symptom severity by sleep quality (A) and sleep duration (B).


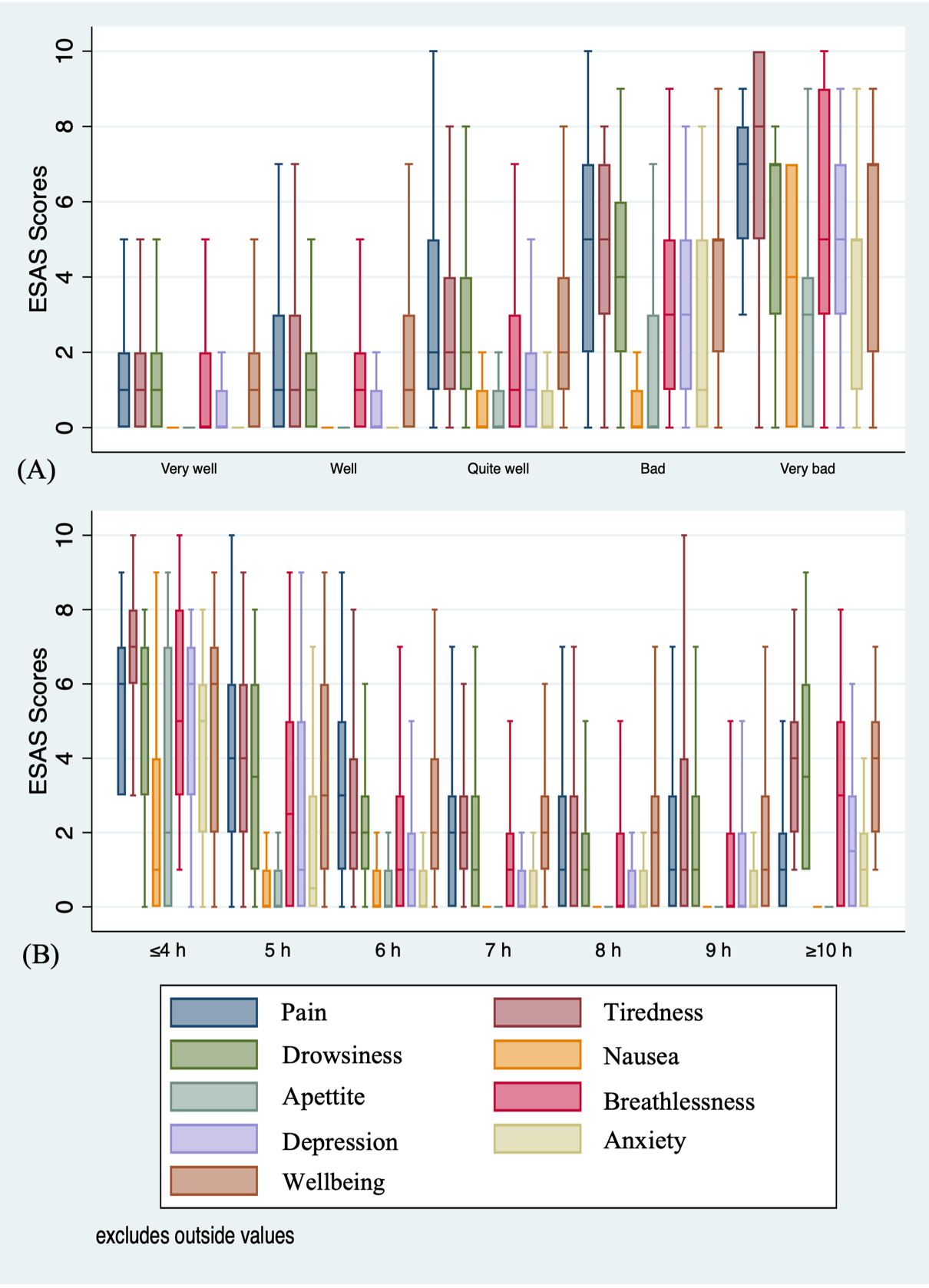

Supplement: online supplemental material 1 [file bmjopen-15-7-s001.docx]
