# Supplementary material for: Association of multiple symptoms with sleep quality and duration: a cross-sectional population-based study of older men in Sweden
Source: BMJ Open. 2025 Jul 1;15(7):e094962. doi: 10.1136/bmjopen-2024-094962 (PMC12215098; doi:10.1136/bmjopen-2024-094962)
Supplement: Supplementary file 2 [file bmjopen-2024-094962.reviewer_comments.pdf]

PEER REVIEW HISTORY

BMJ Open publishes all reviews undertaken for accepted manuscripts. Reviewers are asked to complete a checklist review form and are provided with free text boxes to elaborate on their assessment. These free text comments are reproduced below.

ARTICLE DETAILS

Title (Provisional)

The association of multiple symptoms with sleep quality and duration: a cross-sectional population-based study of older men in Sweden.

Authors

Zeaiter, Maria; Olsson, Max; Kochovska, Slavica; Currow, David; Ekström, Magnus

---

VERSION 1 - REVIEW

**Reviewer**	**1**
**Name**	**Weinreich, G**
**Affiliation**	**Ruhrlandklinik Klinik fur Pneumologie, Pulmology**
**Date**	**13-Nov-2024**
**COI**	**None**

The authors Maria Zeaiter et al present the study "Multiple symptoms are related to worse sleep quality and duration: the population-based VASCOL study of elderly men". This study evaluated any association of the presence and severity of nine major symptoms (pain, tiredness, drowsiness, nausea, appetite, breathlessness, depression, anxiety, and perceived well-being) with sleep quality and duration in elderly men. The authors found that 11% had worse sleep quality, 8% had shorter sleep duration, and 5% had both. Higher severity of each symptom was associated with worse sleep quality (adjusted odds ratios (aOR) ranging 1.12-1.61) and shorter sleep duration (aORs ranging 1.10-1.49). Thus, the authors conclude that A wide range of symptoms are associated with worse sleep quality and shorter sleep duration in elderly men, suggesting that clinicians should assess sleep when these symptoms are present and vice versa. The methods are well-described and the findings are presented appropriately. Please find my questions, comments and concerns below:

1. The authors indicate that the inclusion of only 73-year old men effectively preclude age- or sex-related bias but decrease the generalizability of the findings. Another point of a decrease of generalizability is – it seems to me – that the prevalence of some diseases in this cohort is very low. For instance, in the general population COPD prevalence is about 25% (instead of 3.1% and 5.7%), asthma is about 20% (instead of 5.1% and 8.0%), and sleep

apnoea is about 50% (instead of 7.2% and 20.5%). I would like to ask the authors to discuss these comparable lower prevalences and it's impact on the generalizability.

2. It is surprising that in this age group only 11% report worse sleep quality. I would like to ask the authors to discuss this issue.

3. I would like to ask the authors whether it is possible to provide the number of sleep apnoea patients with CPAP or other therapies.

4. Maybe the sleep apnoea patients in the group "better sleep quality" have CPAP or other therapies. I would like to ask the authors whether it is possible to deliver information on that.

5. In general, it can be assumed that sleep quality is dependent on therapy adherence as well. Do the authors have any data whether subjects took their medication regularly or not?

Reviewer 2

Name Itani, Osamu

Affiliation International University of Health and Welfare, Division of Public Health, School of Medicine

Date 03-Jun-2025

COI None

I understand that this is a cross-sectional epidemiological study examining the relationship between major symptoms observed in elderly Swedish men and sleep quality or duration.

What I found unique about this study is that it focused on the relationship between sleep and indicators evaluated by a collection of major symptoms in various elderly individuals. The study design, analytical methods, interpretation of results, and discussion are excellent. I believe the study is ready for publication in an international journal.

To further enhance the value of this study, I have the following suggestions.

Minor Points:

Please include all P-values in Table 1 up to the third decimal place. Ex. 0.045 <0.001 0.250

Table 2 does not include P-values; however, please include all p-values up to the third decimal place.

The formatting of the References section differs slightly from the journal's submission guidelines. Revise the References section to comply with the submission guidelines using the following example as a reference.

Ex. (from BMJ guidelines)

Journals:

Print journal article: Koziol-Mclain J, Brand D, Morgan D, et al. Measuring injury risk factors: question reliability in a statewide sample. Inj Prev 2000;6:148–50.

Online only journal article: Dark P, Dunn G, Chadwick P, et alThe clinical diagnostic accuracy of rapid detection of healthcare-associated bloodstream infection in intensive care using multipathogen real-time PCR technology. BMJ Open 2011;1:e000181. doi: 10.1136/bmjopen-2011-000181

Supplement article: Mugosa A, Cizmovic M, Lakovic T, et alAccelerating progress on effective tobacco tax policies in Montenegro. Tobacco Control 2020;29:s293-s299

Abstract article: Bricca A, Swithenbank Z, Scott N, et al21 Predictors of recruitment in randomised controlled trials of smoking cessation: meta-regression analyses from the IC-SMOKE systematic review project. Abstract competing for the 'doug altman scholarship'. BMJ Evidence-Based Medicine 2019;24:A52-A53.

Rapid response to an article: Krishnamoorthy KM, Dash PK. Novel approach to transseptal puncture. Heart Online [Rapid response] 18 September 2001. http://heart.bmj.com/cgi/eletters/86/5/e11#EL1

Databases and websites:

Preprints: Rostami A, Sepidarkish M, Leeflang M, et al. First snap-shot meta-analysis to estimate the prevalence of serum antibodies to SARS-CoV-2 in humans. MedRxiv 20185017 [Preprint]. September 02, 2020 https://doi.org/10.1101/2020.08.31.20185017.

Data citations: Wang G, Zhu Z, Cui S, at al. Glucocorticoid induces incoordination between glutamatergic and GABAergic neurons in the amygdala. Dryad Digital Repository [dataset]. August 11, 2017. https://doi.org/10.5061/dryad.k9q7h.

Electronic citations: Moore A. Paracetamol: widely used and largely ineffective [online]. 2018. http://uk.cochrane.org/news/paracetamol-widely-used-and-largely-ineffective (accessed 23 May 2018).

Books and Legal:

Book: Howland J. Preventing Automobile Injury: New Findings From Evaluative Research. Dover, MA: Auburn House Publishing Company 1988:163–96.

Chapter in a book: Nagin D. General deterrence: a review of the empirical evidence. In: Blumstein A, Cohen J, Nagin D, eds. Deterrence and Incapacitation: Estimating the Effects of Criminal Sanctions on Crime Rates. Washington, DC: National Academy of Sciences 1978:95–139.

Legal material: Toxic substances Contro Act: Hearing on S776 Before the Subcommittee of the Environment of the Senate Comm. on Commerce, 94th Congress 1st September (1975).

Law references: The two main series of law reports, Weekly Law Reports (WLR) and All England Law Reports (All ER) have three volumes a year e.g. Robertson v Post Office [1974] 1 WLR 1176

VERSION 1 - AUTHOR RESPONSE

Reviewer: 1
Dr. G Weinreich, Ruhrlandklinik Klinik fur Pneumologie

Comments to the Author:
The authors Maria Zeaiter et al present the study "Multiple symptoms are related to worse sleep quality and duration: the population-based VASCOL study of elderly men". This study evaluated any association of the presence and severity of nine major symptoms (pain, tiredness, drowsiness, nausea, appetite, breathlessness, depression, anxiety, and perceived well-being) with sleep quality and duration in elderly men. The authors found that 11% had worse sleep quality, 8% had shorter sleep duration, and 5% had both. Higher severity of each symptom was associated with worse sleep quality (adjusted odds ratios (aOR) ranging 1.12-1.61) and shorter sleep duration (aORs ranging 1.10-1.49). Thus, the authors conclude that A wide range of symptoms are associated with worse sleep quality and shorter sleep duration in elderly men, suggesting that clinicians should assess sleep when these symptoms are present and vice versa. The methods are well-described and the findings are presented appropriately. Please find my questions, comments and concerns below:

Reply: We thank reviewer 1 for the valuable feedback that we think improved the manuscript. Please see our point-by-point replies below.

1. The authors indicate that the inclusion of only 73-year old men effectively preclude age- or sex-related bias but decrease the generalizability of the findings. Another point of a decrease of generalizability is – it seems to me – that the prevalence of some diseases in this cohort is very low. For instance, in the general population COPD prevalence is about 25% (instead of 3.1% and 5.7%), asthma is about 20% (instead of 5.1% and 8.0%), and sleep apnoea is about 50% (instead of 7.2% and 20.5%). I would like to ask the authors to discuss these comparable lower prevalences and it's impact on the generalizability.

Reply: Thank you for pointing this out. We have added the following text in the Discussion at page 14 to bring up how this can impact the generalizability of the findings of the study: *"The cohort was overall healthy with low prevalence of chronic diseases*

and is comparable to older males in Sweden [1] but the generalisability of our findings could be worse for populations with a higher prevalence of chronic diseases. People with chronic diseases are probably less likely to participate in studies and there is there for a risk that we underestimated the burden of worse sleep quality and short sleep duration in the older general population."

2. It is surprising that in this age group only 11% report worse sleep quality. I would like to ask the authors to discuss this issue.

Reply: We have now added the following text in the discussion on page 14 to discuss this: "*Compared to previous studies of sleep quality among older people, [2,3] our study had a low prevalence of participants with sleep problems that could be explained by our overall healthy cohort[1] but also by different measurements and definitions of worse sleep quality."*

3. I would like to ask the authors whether it is possible to provide the number of sleep apnoea patients with CPAP or other therapies.

Reply: This is a good suggestion, but unfortunately, we don't have the data on CPAP or other therapies for sleep apnoea.

4. Maybe the sleep apnoea patients in the group "better sleep quality" have CPAP or other therapies. I would like to ask the authors whether it is possible to deliver information on that.

Reply: Please see our previous reply.

5. In general, it can be assumed that sleep quality is dependent on therapy adherence as well. Do the authors have any data whether subjects took their medication regularly or not?
Reply: This would be interesting but unfortunately we don't have data on this.

Reviewer: 2
Prof. Osamu Itani, International University of Health and Welfare

Comments to the Author:
I understand that this is a cross-sectional epidemiological study examining the relationship between major symptoms observed in elderly Swedish men and sleep quality or duration.
What I found unique about this study is that it focused on the relationship between

sleep and indicators evaluated by a collection of major symptoms in various elderly individuals. The study design, analytical methods, interpretation of results, and discussion are excellent. I believe the study is ready for publication in an international journal.
To further enhance the value of this study, I have the following suggestions.
Reply: We thank reviewer 2 for this positive feedback and have now revised the manuscript accordingly. See our point-by-point replies below.

Minor Points:
Please include all P-values in Table 1 up to the third decimal place. Ex. 0.045 <0.001 0.250
Table 2 does not include P-values; however, please include all p-values up to the third decimal place.

Reply: We have now changed to three decimal places for all p-values in Table 1 and added p-values to Table 2 with three decimal places.

The formatting of the References section differs slightly from the journal's submission guidelines. Revise the References section to comply with the submission guidelines using the following example as a reference.

Ex. (from BMJ guidelines)
Journals:
Print journal article: Koziol-Mclain J, Brand D, Morgan D, et al. Measuring injury risk factors: question reliability in a statewide sample. Inj Prev 2000;6:148–50.
Online only journal article: Dark P, Dunn G, Chadwick P, et alThe clinical diagnostic accuracy of rapid detection of healthcare-associated bloodstream infection in intensive care using multipathogen real-time PCR technology. BMJ Open 2011;1:e000181. doi: 10.1136/bmjopen-2011-000181
Supplement article: Mugosa A, Cizmovic M, Lakovic T, et alAccelerating progress on effective tobacco tax policies in Montenegro. Tobacco Control 2020;29:s293-s299
Abstract article: Bricca A, Swithenbank Z, Scott N, et al21 Predictors of recruitment in randomised controlled trials of smoking cessation: meta-regression analyses from the IC-SMOKE systematic review project. Abstract competing for the 'doug altman scholarship'. BMJ Evidence-Based Medicine 2019;24:A52-A53.
Rapid response to an article: Krishnamoorthy KM, Dash PK. Novel approach to transseptal puncture. Heart Online [Rapid response] 18 September 2001. http://heart.bmj.com/cgi/eletters/86/5/e11#EL1
Databases and websites:

Preprints: Rostami A, Sepidarkish M, Leeflang M, et al. First snap-shot meta-analysis to

estimate the prevalence of serum antibodies to SARS-CoV-2 in humans. MedRxiv 20185017 [Preprint]. September 02, 2020 https://doi.org/10.1101/2020.08.31.20185017.
Data citations: Wang G, Zhu Z, Cui S, at al. Glucocorticoid induces incoordination between glutamatergic and GABAergic neurons in the amygdala. Dryad Digital Repository [dataset]. August 11, 2017. https://doi.org/10.5061/dryad.k9q7h.
Electronic citations: Moore A. Paracetamol: widely used and largely ineffective [online]. 2018. http://uk.cochrane.org/news/paracetamol-widely-used-and-largely-ineffective (accessed 23 May 2018).
Books and Legal:

Book: Howland J. Preventing Automobile Injury: New Findings From Evaluative Research. Dover, MA: Auburn House Publishing Company 1988:163–96.
Chapter in a book: Nagin D. General deterrence: a review of the empirical evidence. In: Blumstein A, Cohen J, Nagin D, eds. Deterrence and Incapacitation: Estimating the Effects of Criminal Sanctions on Crime Rates. Washington, DC: National Academy of Sciences 1978:95–139.
Legal material: Toxic substances Contro Act: Hearing on S776 Before the Subcommittee of the Environment of the Senate Comm. on Commerce, 94th Congress 1st September (1975).
Law references: The two main series of law reports, Weekly Law Reports (WLR) and All England Law Reports (All ER) have three volumes a year e.g. Robertson v Post Office [1974] 1 WLR 1176

Reply: We have now changed the references according to the submission guidelines.

We would ones again thank the reviewers for the valuable feedback that we think improved the manuscript.

Yours sincerely,

Max Olsson, PhD, Lund University

On behalf of the authors.

REFERENCES

1. Olsson M, Engström G, Currow DC, Johnson M, Sandberg J, Ekström MP. VAScular and Chronic Obstructive Lung disease (VASCOL): a longitudinal study on morbidity, symptoms and quality of life among older men in Blekinge county, Sweden. *BMJ Open* 2021; **11**(7): e046473.

2. Suzuki K, Miyamoto M, Hirata K. Sleep disorders in the elderly: Diagnosis and management. *J Gen Fam Med* 2017; **18**(2): 61-71.
3. Fernandes M, Antonucci M, Capecci F, Mercuri NB, Della-Morte D, Liguori C. Prevalence of sleep disorders in geriatrics: an exploratory study using sleep questionnaires. *Geriatric Nursing* 2024; **60**: 107-13.
